# Rapid Detection and Quantification of Viable Cells of *Pectobacterium brasiliense* Using Propidium Monoazide Combined with Real-Time PCR

**DOI:** 10.3390/microorganisms11112808

**Published:** 2023-11-19

**Authors:** Junhui Li, Ruxing Chen, Ruwei Yang, Xinchen Wei, Hua Xie, Yanxia Shi, Xuewen Xie, Ali Chai, Tengfei Fan, Baoju Li, Lei Li

**Affiliations:** 1 State Key Laboratory of Vegetable Biobreeding, Institute of Vegetables and Flowers, Chinese Academy of Agricultural Sciences, Beijing 100081, China; rancho_lee@yeah.net (J.L.); kuutamo@yeah.net (R.C.); weixinchen2022@163.com (X.W.); shiyanxia@caas.cn (Y.S.); xiexuewen@caas.cn (X.X.); chaiali@caas.cn (A.C.); fantengfei@caas.cn (T.F.); 2 Comprehensive Experimental Farm, Xinjiang Academy of Agricultural Sciences, Urumqi 830091, China; yangruwei@yeah.net; 3 Beijing Agro-Biotechnology Research Center, Beijing Academy of Agriculture and Forestry Sciences, Beijing 100097, China; xiehua@baafs.net.cn

**Keywords:** *Pectobacterium brasiliense*, bacterial soft rot, PMA-qPCR, detection, viable cell

## Abstract

*Pectobacterium brasiliense* (*Pbr*) has caused significant economic losses in major vegetable production areas in Northern China by causing bacterial soft rot in cash crops such as potatoes and cucumbers. This study aimed to establish a PMA-qPCR detection method for *Pbr* by screening specific and sensitive primers based on the *glu* gene and the conserved region of the 23S rRNA gene. Based on the optimized PMA pretreatment conditions, a standard curve was designed and constructed for PMA-qPCR detection (y = −3.391x + 36.28; R^2^ = 0.99). The amplification efficiency reached 97%, and the lowest detection limit of viable cells was approximately 2 × 10^2^ CFU·mL^−1^. The feasibility of the PMA-qPCR method was confirmed through a manually simulated viable/dead cell assay under various concentrations. The analysis of potato tubers and cucumber seeds revealed that nine naturally collected seed samples contained a range from 10^2^ to 10^4^ CFU·g^−1^ viable *Pbr* bacteria. Furthermore, the system effectively identified changes in the number of pathogenic bacteria in cucumber and potato leaves affected by soft rot throughout the disease period. Overall, the detection and prevention of bacterial soft rot caused by *Pbr* is crucial.

## 1. Introduction

Bacterial soft rot has been considered one of the most destructive bacterial diseases in greenhouse vegetable production, affecting an estimated 15–20% of global crop production and exhibiting an aggravating annual trend [1,2,3]. *Pectobacterium brasiliense* (*Pbr*) is the causative pathogen of bacterial soft rot, and this disease causes the maceration of plant tissue, the appearance of water-soaked lesions, and the collapse of infected tissue, which ultimately causes the plant to wither and die. This disease has been reported in Eurasia, Africa, and South Africa [4,5,6], and especially in China, where bacterial soft rot caused by *Pbr* has become a devastating disease, resulting in a 30% reduction in potato and cucumber production across numerous provinces [7,8,9,10]. With the lack of commercial cultivars resistant to bacterial soft rot, disease control in production settings, including seed production, has heavily relied on the application of bactericides, but the efficacy of these applications is inconsistent and generally low, and the continuous use of an antibiotic elevates the drug resistance exhibited by pathogenic bacteria [2,11]. Contaminated or infected tissue from seeds and plant debris is considered the main source of soft rot inoculum [12,13]. Detecting *Pbr* at an early stage can greatly aid the prevention of outbreaks, as seed transmission remains one of the primary methods by which a plant becomes infected with this disease [8,14,15].

Commercial seeds are often treated to reduce contamination with surface pathogens, but these bacterial pathogens are often present in varying amounts depending on the treatment method. To limit the spread of infected seeds and plant debris and reduce the inoculum size, highly sensitive and specialized detection tools are needed to detect the presence of the pathogen [16,17,18]. The traditional bacterial detection methods require culturing microorganisms on agar plates, which is time-consuming and labor-intensive [19,20]. However, the sensitivity of this method exhibits significant inaccuracies, as microorganisms in diseased seeds or plant tissues compete with or inhibit the growth of the target pathogen. Although a few reverse-transcription polymerase chain reaction (RT-PCR) studies have investigated *Pectobacterium* species [21,22], *Pbr* has not been studied.

Moreover, the majority of these assays do not have the ability to distinguish between DNA extracted from live or deceased bacterial cells, leading to the possibility of producing inaccurate positive outcomes. One such assay is the biological PCR assay, which includes a nutrient enrichment step to enhance the sensitivity of PCR to detect DNA from live pathogen cells, but biological PCR assays also suffer from the aforementioned culture limitations. Given the enormous impact of bacterial soft rot caused by *Pbr* on the in-greenhouse vegetable production industry [7,23,24,25], it is important to be able to selectively detect active pathogen cells using more sensitive and specific assays.

In recent years, in vivo quantitative PCR (qPCR) methods that use DNA-intercalating dyes to avoid DNA amplification in dead cells of target pathogens have become popular for the selective quantification of DNA in living cells. One of the most commonly used dyes in live qPCR assays is propidium monoazide (PMA), which, due to its membrane damage, can penetrate dead cells and, upon photoactivation, covalently bind to DNA in these cells [26]. Dye cross-linking prevents the amplification of dead cell DNA during PCR detection. Unbound excess dye is photo-inactivated to prevent its interference with the PCR amplification of DNA extracted from living cells. The PMA-qPCR detection method has been employed for various plant pathogenic bacteria [3,27,28,29,30] but not for *Pbr*.

The present study aimed to assess the combined use of PMA and qPCR for differentiating between active and inactive *Pbr* in plant tissues and seeds. The objectives were as follows: (i) design and validation of specific primers based on the 23S rRNA gene sequences of the *glu* and *Pbr* genes; (ii) optimization of PMA pretreatment conditions and construction of a standard curve; and (iii) detection of infectious *Pbr* in naturally contaminated potato and cucumber leaves, tissues, or seeds using an established PMA-qPCR method. The results provide a scientific basis for the development of new molecular antibacterial agents against cucumber soft rot, the effective control of the threat of plant soft rot, and the control and early diagnosis of disease caused by pathogenic bacteria.

## 2. Materials and Methods

### 2.1. Bacterial Strains, Cultivation, and DNA Extraction

In order to evaluate the specificity of the primers, a total of 48 strains of various *Pectobacterium* spp., including SX309, and other bacteria that are known to cause bacterial soft rot were employed for specificity testing (Table 1). The strains needed for the test were inoculated onto Luria–Bertani (LB) solid medium and incubated at 28 °C for 24 h. The strains were diluted to an OD600 of approximately 1.0 and then set aside.

### 2.2. Primer Design and Specificity Testing

**Figure 1:**
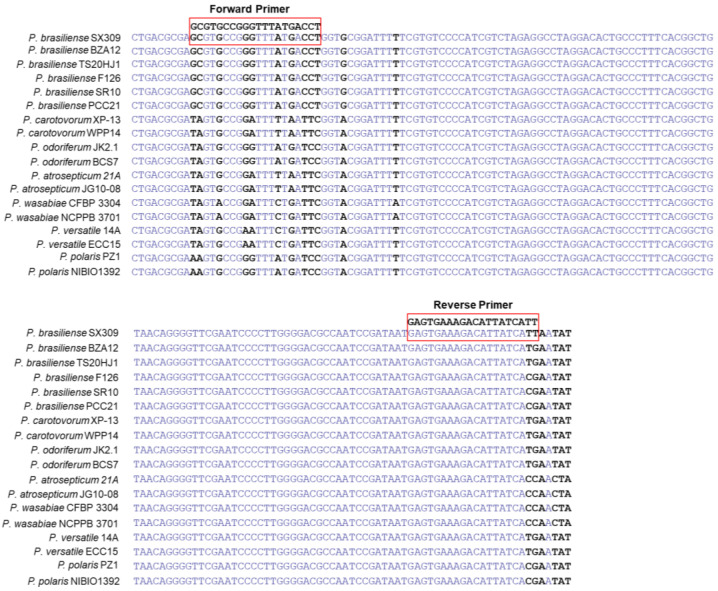
Alignment indicating the positions of the designed *Pectobacterium brasiliense* primers and probes on the 16-23S intergenic spacer region of ribosomal RNA and the tRNA-Glu nucleotide sequence in comparison to other *Pectobacterium* species.

The NCBI database was used to search for DNA homologues based on comparison of the sequences of the *glu* and 23S rRNA genes in *Pbr* with those in other species of *Pectobacterium* with the aims of finding partial sequences with differences in their conserved regions (Table 1 and Figure 1) and designing primers specific to *Pbr* based on these partial sequences. The primer pair PMA1-F (5′-GCGTGCCGGGTTTATGACCT-3′) and PMA1-R (5′-AATGATAATGTCTTTCACTC-3′) was designed for the specific amplification of *Pbr*. The strains used to investigate the specificity of the primer are listed in Table 1. PCR was employed to amplify the products, and the reaction procedure was as follows: 95 °C for 10 min, 30 cycles of 95 °C for 15 s, 50 °C for 20 s, 72 °C for 10 s, and 72 °C for 10 min. The amplification products of *Pbr* SX309 were resequenced, and the identities of the sequences were checked against the NCBI database.

### 2.3. Primer Sensitivity Testing and Quantification Standards

The confirmed *Pbr* SX309 product was purified, ligated to the pEASY-T1 vector (TransGen Biotech, Beijing, China), and transformed into DH5α cells (9057, Takara, Dalian, China). The cells were then inoculated onto Luria–Bertani plates containing 50 μL·mL^−1^ of ampicillin and incubated overnight at 37 °C to obtain plasmids. Individual positive plaques were picked and sequenced for comparison. The plasmid concentrations were measured using a Tnano-800 ultramicroscopic spectrophotometer (Tuohe, Shanghai, China), and the copy number of the plasmid was calculated according to Fronhoffs [31]. Gradient dilutions were performed to generate templates for fluorescent quantitative PCR.

A standard curve was created to measure *Pbr* by analyzing a 10-fold dilution series of the plasmids. Plasmids quantities ranging from 2.24 × 10^7^ to 2.24 × 10^1^ copies·µL^−1^ were prepared using sterile distilled water. qPCR was conducted using a reaction volume of 20 µL, which consisted of 1 µL of plasmid DNA, 10 µL of SuperReal PreMix Plus (FP215, TIANGEN, Beijing, China), and 0.4 µL of each primer (PMA1-F/PMA1-R). The real-time PCR system used was an ABI 7000 instrument (Thermo Fisher, Waltham, MA, USA) under the following cyclic conditions: 95 °C for 60 s, followed by 40 cycles of 95 °C for 5 s, and 60 °C for 31 s. The cycle threshold (Ct) values were automatically calculated using ABI 7500 software (Real-Time PCR Software v2.4). The detection limit of qPCR was defined as the lowest concentration at which the replicates revealed a Ct ≤ 35 [32].

### 2.4. Optimization of the PMA Pretreatment Concentration

The optimization of the pretreatment conditions for the quantitative detection of viable *Pbr* using PMA-qPCR involved establishing different PMA pretreatment concentrations. The bacterial suspensions were pretreated with different concentrations of PMA, and the optimal concentration to penetrate into dead cells as opposed to live cells was determined via fluorescent quantitative PCR. *Pbr* SX309 was activated and purified before picking single colonies in NB liquid medium. The colonies were incubated in a shaker at 180 rpm for 24 h at 28 °C. The bacterial suspension concentration was then adjusted to an OD_600_ of 1 corresponding to 10^8^ CFU·mL^−1^ and diluted to 10^7^ CFU·mL^−1^ and 10^6^ CFU·mL^−1^ using sterilized water. Each bacterial suspension concentration was divided into two groups: group A was maintained in an ice bath, and group B was inactivated at 95 °C for 20 min. Group A was used as a live cell suspension, while group B was used as a dead cell suspension.

The final concentrations of PMA were then adjusted to 0 μmol·L^−1^, 10 μmol·L^−1^, 20 μmol·L^−1^, 40 μmol·L^−1^, 60 μmol·L^−1^, 80 μmol·L^−1^, and 100 μmol·L^−1^ and mixed with 200 μL of bacterial suspensions containing different concentrations (10^8^ CFU·mL^−1^, 10^7^ CFU·mL^−1^, and 10^6^ CFU·mL^−1^) of cells. The reactions were completely reacted in the dark for 10 min, and the reacted bacterial suspensions were then treated under a 50 W LED light for 5 min. Three samples of group A and three samples of group B were obtained under different concentrations of PMA. This process was conducted in an ice bath to avoid high temperatures. The experimental treatment was replicated three times.

Two hundred microliters of the *Pbr* strain mixture, which was pretreated with PMA, was used for the extraction of 20 μL of DNA using the bacterial genomic DNA extraction kit (TIANGEN, DP302-02, Beijing, China). The extraction process was carried out according to the manufacturer’s instructions. Subsequently, Ct values were determined using the qPCR method from six different groups. The experimental treatment was replicated three times.

### 2.5. The PMA-qPCR Assay Using Different Ratios of Viable and Dead Cells

The ability of the PMA-qPCR method to differentiate between live and dead cells was assessed and verified. To this end, a mixture of varying percentages of live and dead cells was utilized to gauge the reliability of this method. To obtain mixed suspensions of viable/dead *Pbr* bacteria under varying concentrations, colonies were selected from an activated *Pbr* SX309 strain and then incubated in 150 mL of NB liquid medium at 28 °C and 200 rpm for 12 h. After incubation, the bacteria were diluted to a concentration of 1 × 10^7^ CFU·mL^−1^. Equal amounts of the suspensions were subjected to high temperatures and refrigeration, which revealed that these suspensions were inactivated. Multiple mixtures of live and dead cells were generated with varying percentages of live cells (0.01%, 0.1%, 1%, 10%, 50%, and 100%) and were subsequently analyzed using different methods, such as plate coating, fluorescence quantitative PCR, and PMA-qPCR. Each mixture was replicated three times and processed via plating on medium for (1) CFU counting, (2) treatment with 80 μmol·L^−1^ of PMA, DNA extraction and real-time PCR, and (3) direct DNA extraction without PMA treatment and tracking via real-time PCR. After DNA extraction, the PMA-qPCR assay system was used to quantify pathogenic *Pbr* bacteria. For each treatment, 0.2 mL of the mixture was used, and this experiment was replicated three times.

### 2.6. Analysis of Artificially Contaminated Cucumber Seeds via the PMA-qPCR Assay

In order to artificially obtain *Pbr*-contaminated cucumber seeds, the seeds were first sterilized by soaking in a 1% sodium hypochlorite solution for 5 min and then rinsed three times with sterile water. The seeds were then air-dried to avoid direct exposure to light. One hundred seeds were then added to 100 mL of a bacterial suspension within a 10-fold gradient from 10^8^ CFU·mL^−1^ to 10^2^ CFU·mL^−1^. Thirty minutes after inoculation, the seeds were air-dried under room temperature. DNA was extracted from 10 seeds randomly collected from each treatment, and a live cell assay was then performed based on the constructed PMA-qPCR method.

### 2.7. Analysis of Pbr Mortality in Cucumber Seeds after Artificial Warm Broth Soaking Based on the PMA-qPCR Assay

Cucumber seeds impregnated with the *Pbr* bacterial suspension were treated in a water bath at the temperatures of 50 °C, 55 °C, and 60 °C for 10 min, 30 min, and 60 min using the warm broth method. In this study, the number of cucumber seeds per treatment was 100, and the concentration of each *Pbr* fermentation broth used was 1 × 10^6^ CFU·mL^−1^. Each set of treatments had three replicates. The lethality of the pathogen in cucumber seeds was then determined via the PMA-qPCR method.

The mortality rate of the pathogens was calculated using the following formula: T = (N − Nt)/N × 100%, where N is the quantity of viable pathogenic cells in the seeds, and Nt is the number of viable pathogenic cells in the seeds treated under the respective temperature.

### 2.8. Detection of Viable Pbr in Potato Tuber and Cucumber Seed Samples Collected from Different Regions of China Based on the PMA-qPCR Method

Fifteen healthy-looking potato tubers were randomly collected from different potato-producing areas in China and selected for analysis of the occurrence of potato soft rot disease. After washing off the soil on the surface of potato tubers with sterile water, 2 g of potato skin (thickness of approximately 2 mm) was scraped, ground with liquid nitrogen, and added to 10 mL of sterile water. Subsequently, 0.5 mL of the mixture was sucked up for the PMA-qPCR assay, and the number of viable PBR copies per gram of the samples was calculated. Each sample was assessed three times.

Forty groups of cucumber seeds were collected from cucumber-growing areas in Northern China that were contaminated with different levels of *Pbr* under natural conditions. Two grams of seeds were pulverized with a mortar and pestle. Ten milliliters of sterile water were then added to create a white homogeneous suspension, and the seed residue was filtered. The resulting filtrate was pretreated with the optimized PMA condition, and 0.5 mL of it was collected for DNA extraction and used in a fluorescent quantitative PCR assay to detect any potential pathogens present in the seeds. Each sample was assessed three times.

### 2.9. Dynamic Changes in Viable Pbr Amounts on Cucumber and Potato Leaves Detected via the PMA-qPCR Assay

The PMA-qPCR technique was employed to monitor the proliferation of living *Pbr* cells in the tissues of *Pbr*-infected cucumber and potato leaves during disease progression. The test subjects for the experiment were one-month-old potato and cucumber seedlings at the 2–3 true leaf stages. The bacterial suspension with a concentration of 1 × 10^8^ CFU·mL^−1^ was uniformly sprayed on the leaves of the experimental group using a microsprayer, whereas the control group was sprayed with sterile water. Ten plants from each group were selected, and the experiment was replicated three times. The inoculated cucumbers and potatoes were incubated in an artificial climate chamber at 28 °C with 85% relative humidity. Cucumber leaves were randomly sampled at 0 h, 12 h, 24 h, 36 h, and 48 h post-treatment. Five leaves were randomly selected at each time point, cut, ground, and mixed. Following PMA treatment, 0.2 g of tissue solution was extracted, and the process was repeated three times for each group. The extracted DNA was detected using the PMA-qPCR detection system, which quantified the pathogens, and the number of viable bacteria was calculated based on the Ct value, copy number, and dilution.

### 2.10. Statistical Analysis

The experiments described in this study were established with three sets of experimental replicates. SPSS 17.0 software (IBM, Armonk, New York, NY, USA) was employed for all statistical analyses. One-way analysis of variance (ANOVA) with a significance level of *p* < 0.05 was conducted to detect significant differences in the means. The Ct and DI values were expressed as the means ± SDs.

## 3. Results

### 3.1. Specificity of the Primers

**Figure 2:**
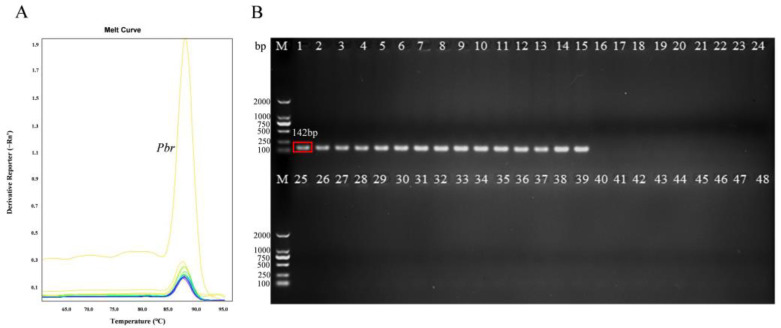
qPCR solubility curve (**A**) and electrophoresis graph of amplification products (**B**) for *Pbr* and other species strains. Notes: M: 2000 bp Maker. The strain numbers are listed in Table 1.

Based on the conserved sequences of *Pbr* strains, several differences were observed in a number of loci between the *glu* and 23S rRNA genes of various *Pectobacterium* species (Figure 1). The amplification of purified *Pbr* DNA using the primers PMA1-F and PMA1-R produced a 142-bp PCR product (Figure 2). After optimization, the optimal annealing temperature for PCR was 58 °C. These primers specifically target SX309 for PCR amplification in a general PCR assay, and the PCR product produced a 142-bp band with no primer dimerization or non-specific amplification. The analysis of the amplification sequence revealed a complete similarity of 100% with the *Pbr* strain accession sequences in GenBank, which confirmed the accurate amplification of the correct product (Table S1). In addition, real-time PCR revealed that these primers worked optimally under an annealing temperature of 58 °C. The melting curve of *Pbr* SX309 displayed a single peak, with a Tm value of 86.5 °C (Figure 2). These results indicate that this primer can be used to detect *Pbr* via qPCR.

The finding that all 15 strains of *Pbr* from different hosts, including cucumber, Chinese cabbage, celery, and bok choy, could be specifically amplified using this primer demonstrates the inclusiveness of this primer (Table 1 and Figure 2B). Interestingly, twenty-six strains of *Pectobacterium* from other genera collected from different hosts and seven strains of pathogens that cause other common bacterial diseases were not able to be specifically amplified, which further indicated the specificity of this primer (Table 1).

### 3.2. Sensitivity and Standard Curve of Real-Time PCR

**Figure 3:**
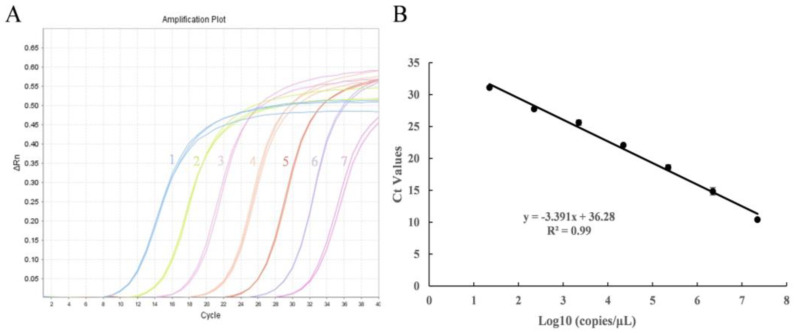
qPCR amplification curves (**A**) and standard curves (**B**) for the copy numbers of gradient-diluted standard plasmids. (**A**) The amplification curves represent the copy number in the range from 2.24 × 10^7^ copies·μL^−1^ to 2.24 × 10^1^ copies·μL^−1^. Dilution gradients are indicated by the number range from 1 to 7. Note: No.1: 2.24 × 10^7^ copies·μL^−1^, No.2: 2.24 × 10^6^ copies·μL^−1^, No.3: 2.24 × 10^5^ copies·μL^−1^, No.4: 2.24 × 10^4^ copies·μL^−1^, No.5: 2.24 × 10^3^ copies·μL^−1^, No.6: 2.24 × 10^2^ copies·μL^−1^, No.7: 2.24 × 10^1^ copies·μL^−1^. (**B**) The logarithms of the plasmid copy numbers were plotted against the Ct values, and the regression line equation and correlation coefficient (R^2^) are displayed. The error bars represent the standard deviations of three replicate reactions.

A dilution series of the *Pbr* plasmid ranging from 2.24 × 10^7^ copies·µL^−1^ to 2.24 × 10^1^ copies·µL^−1^ was used to create a quantification standard curve (Figure 3A). The correlation between the log copies of the *Pbr* plasmid and the corresponding Ct values was observed, as demonstrated via a linear relationship (y = −3.391x + 36.28; R^2^ = 0.99) (Figure 3B). The primer pair PMA1-F/PMA1-R displayed both specificity and efficiency, achieved an amplification efficiency of 97.2%, and permitted accurate quantifications of *Pbr*.

### 3.3. Optimization of PMA Pretreatment Conditions

**Figure 4:**
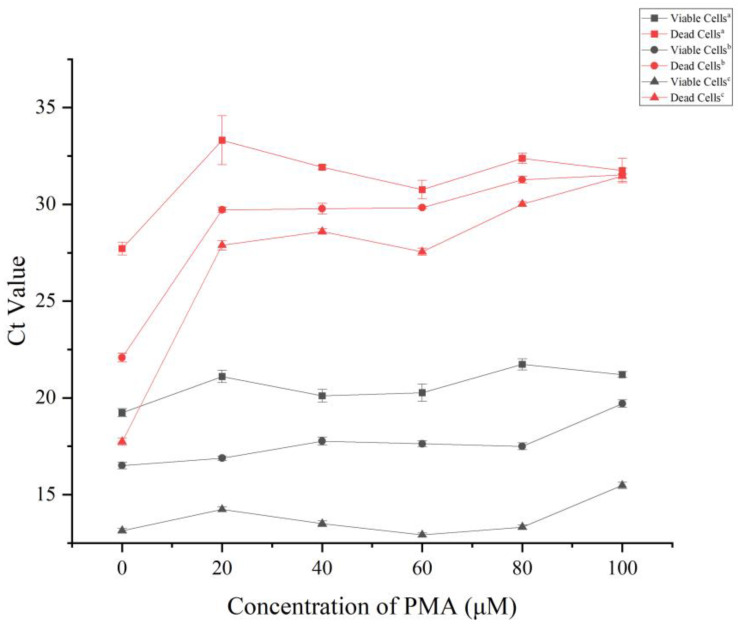
Ct values of viable or dead cells of the *Pbr* strain treated with different concentrations of PMA. Note: ^a^, 10^6^ CFU·mL^−1^; ^b^, 10^7^ CFU·mL^−1^; and ^c^, 10^8^ CFU·mL^−1^. The data have been presented as the means of three replicates ± standard deviations.

In order to accurately differentiate between living and non-living cells during the PMA pretreatment step, determining the optimal concentration of PMA is essential. The concentration should inhibit the amplification of non-viable cells without affecting the amplification of viable cells during qPCR. Based on this finding, viable and heat-killed cells were subjected to various final concentrations of PMA (0 μmol·L^−1^, 10 μmol·L^−1^, 20 μmol·L^−1^, 40 μmol·L^−1^, 60 μmol·L^−1^, 80 μmol·L^−1^, and 100 μmol·L^−1^). A heat-killed cell concentration of 10^6^ CFU·mL^−1^ and a PMA concentration of at least 20 μmol·L^−1^ yielded a Ct value of dead cells that exceeded 30 (Figure 4). At dead cell concentrations reaching 10^7^ CFU·mL^−1^ or 10^8^ CFU·mL^−1^, the Ct value did not surpass 30 until the PMA concentration was 80 μmol·L^−1^ or higher (Figure 4). The Ct values for viable cells remained stable or slightly elevated with increases in the PMA concentration within the range from 0 μmol·L^−1^ to 100 μmol·L^−1^. However, a significant elevation in Ct values was observed when the PMA concentration exceeded 80 μmol·L^−1^. In summary, a final concentration of 80 μmol·L^−1^ PMA would be optimal to entirely suppress dead cell DNA amplification, while having minimal effects on viable cells.

### 3.4. The PMA-qPCR Viable Cell Assay

In order to assess the sensitivity of the PMA-qPCR assay for the detection of viable cells, this method was compared with the qPCR assay and plate counting to validate the ability of the three methods to enumerate the surviving cells in mixtures of different proportions of surviving and dead *Pbr* cells. Based on the single-copy nature of the PMA-1F/1R amplified primer sequence in the *Pbr* SX309 genome, the number of copies detected was denoted as the number of cells. When the proportion of living cells was 100%, the average Ct value obtained via the PMA-qPCR assay was 6.49, and the copy number was 6.49 log10 (copies·mL^−1^); in contrast, the average Ct value obtained via qPCR was 6.49, and the copy number was 6.27 log10 (copies·mL^−1^). Considering the unavoidable loss of strain DNA during the extraction process, the results obtained with these three methods based on a percentage of surviving cells of 100% were consistent compared with those obtained via conventional plate counting. However, reductions in the percentage of viable cells gradually emphasized the limitations of the qPCR assay for detecting viable cells. The mean Ct values obtained with the qPCR assay were 22.98, 24.11, 22.34, 22.79, and 25.65 for live cell percentages of 50%, 10%, 1%, 0.1%, and 0.01%, respectively. These values did not match the cell numbers obtained via plate counting. However, the mean Ct values obtained with the PMA-qPCR assay developed in this study were 22.87, 25.43, 27.98, 32.21, and 34.73. Remarkably, these values closely aligned with the plate counting results (Table 2).

### 3.5. Detection of Viable Pbr Carried on Potato Tubers and Cucumber Seeds Based on the PMA-qPCR Assay

In order to verify the sensitivity of the PMA-qPCR assay for the detection of *Pbr* pathogens carried on cucumber seeds, artificially infected cucumber seeds, and cucumber seeds collected from various regions where cucumber bacterial soft rot had occurred were subjected to a viable cell assay. Cucumber seeds soaked in a suspension of pathogenic bacteria (ranging from 10^6^ CFU·mL^−1^ to 10^8^ CFU·mL^−1^) were detected via PMA-qPCR (Ct value ≤ 30) with 1.71 × 10^4^ to 8.49 × 10^5^ CFU per gram of seed. (Table 3). These results were consistent with the trend obtained via plate counting, indicating that the PMA-qPCR assay is an effective tool for detecting the seed pathogen *Pbr* and for obtaining an early warning of soft rot development.

Fifteen potato samples were assessed via the PMA-qPCR assay (Table 4), and three samples from Xinjiang, China, two samples from Inner Mongolia, and two samples from Heilongjiang were found to have live PBR pathogens on their surfaces. Among these samples, potatoes from the Xinjiang region carried the highest number of pathogenic bacteria on their surface, and potato tubers that did not exhibit obvious soft rot symptoms still contained approximately 5150 CFU·g^−1^ *Pbr* pathogens on their epidermises.

In addition, based on the accuracy of the detection of artificially infected cucumber seeds, 40 collected cucumber seeds that may have contained the *Pbr* pathogen were tested and analyzed via the PMA-qPCR assay (Table 5). Sample 4 was collected from a cucumber plantation in Liaoning Province in China that had been severely infected by *Pbr* and suffered from severe soft rot disease and had the highest amount of *Pbr*, with an average of 8.27 × 10^4^ CFU of *Pbr* pathogens per gram of seed. In addition, six different cucumber seed samples from the Shandong and Shanxi Provinces in China were also found to have *Pbr* pathogens, and these seeds were infected to varying degrees, with Ct values ranging from 27.39 to 30.11. The remaining 31 seed samples were assessed to determine whether they contain *Pbr* pathogens and were found to be negative for *Pbr* (Ct values ≥ 35). Although some of the seed samples were collected from cucumber production areas that had been affected by soft rot, the presence of *Pbr* was not detected via PMA-qPCR as either their amount was too low to be detected or because the *Pbr* pathogens carried in the seeds were dead cells.

### 3.6. Effect of Warm Broth Soaking on the Activity of Pathogenic Bacteria

**Figure 5:**
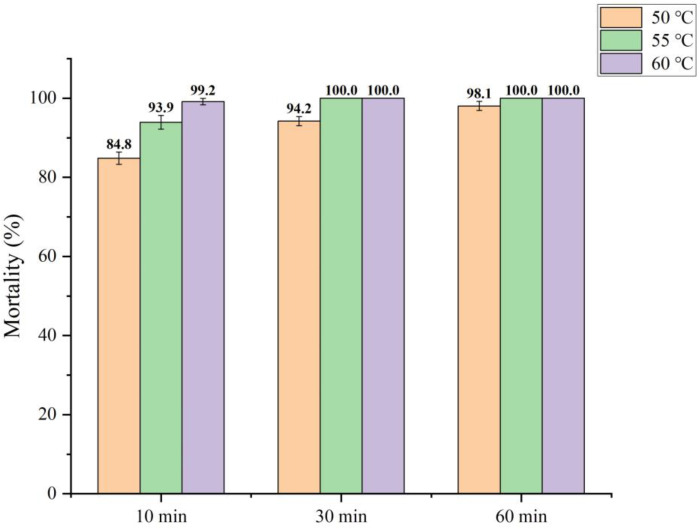
Effects of soaking in warm broth on the mortality of *Pbr* in seeds. The data are presented as the means of three replicates ± standard deviations.

In order to verify the effect of temperature on the activity of pathogenic bacteria in seeds with bacteria, the number of viable cells in seeds after different temperature treatments was detected using the PMA-qPCR method. The results indicated that the lethality of viable pathogenic bacteria in the seeds reached 94% after treatment in a water bath at 50 °C for 10 min, and that the mortality rate reached 100% after 60 min of treatment; the mortality rate of the pathogenic bacteria reached 99% after treatment in a water bath at 55 °C/60 °C for more than 10 min (Figure 5). In summary, the soaking of the seeds in warm broth for 10–30 min at 55 °C~60 °C can effectively eliminate living pathogens from seeds and thus reduce the probability of seeds with bacteria causing disease in the following year.

### 3.7. Dynamic Changes in Pbr on Potato and Cucumber Leaves Detected via PMA-qPCR

**Figure 6:**
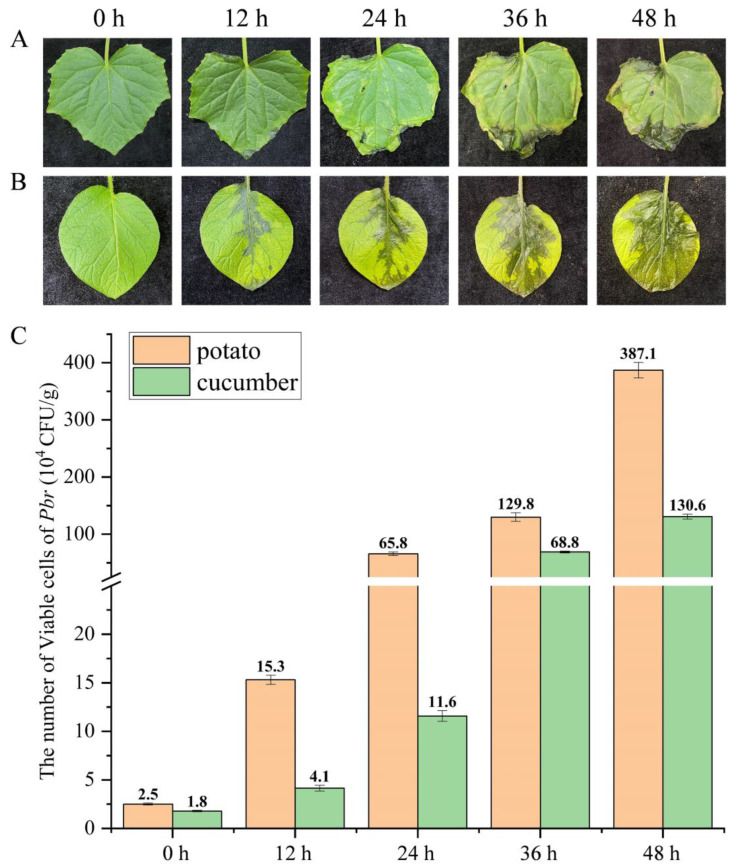
Dynamic lesions and number of viable cells of *Pbr* in cucumber and potato leaves from 0 to 48 h after infection with bacterial soft rot disease. (**A**) Dynamics of cucumber leaves over 48 h. (**B**) Dynamics of potato leaves over 48 h. (**C**) Number of viable *Pbr* bacteria on cucumber and potato leaves detected via PMA-qPCR. The data are presented as the means of three replicates ± standard deviations.

In order to assess the validity of the PMA-qPCR assay for early disease control, cucumber seedlings with 2-3 true leaves and potatoes grown for 1 month were sprayed and inoculated with the pathogen. Disease symptoms at varying degrees were observed on the leaves of cucumbers and potatoes at 0 h, 12 h, 24 h, 36 h, and 48 h after treatment. At 12 h post-inoculation, soft rot symptoms were observed on the leaf margins or petioles, which gradually enlarged in size over time. By 48 h, both cucumber and potato leaves displayed severe rot symptoms (Figure 6A,B). The PMA-qPCR detection system was used to quantify the level of *Pbr* pathogen on leaves at different time points. The results indicated that the initial level of *Pbr* on potato and cucumber leaves was 2.5 × 10^4^ CFU·g ^−1^ and 1.8 × 10^4^ CFU·g^−1^, respectively. Over time, the number of *Pbr* bacteria on potato leaves significantly increased, reaching 3.87 × 10^6^ CFU·g^−1^ per leaf at the 48-h time point (Figure 6C). The trend observed for *Pbr* bacteria on the cucumber leaves was consistent with that observed on the potato leaves, although the cucumber leaves exhibited a slightly lower bacterial load overall. This difference in the bacterial load could be attributed to the variations in the numbers of disease spots on the leaves, which was also indicative of the ability of this method to accurately and reliably detect the number of live *Pbr* cells on the infected plants. Overall, these results suggest that this method can be used to provide crucial insights into the detection and control of disease in infected plants and would thereby aid early disease prevention and treatment.

## 4. Discussion

Bacterial soft rot disease caused by *Pectobacterium* is a highly infectious disease restricting crop production [33,34]. In the absence of effective control agents, early warning and diagnosis of disease are crucial, especially for seed-borne diseases such as bacterial soft rot disease caused by *Pbr* [15], and this depends on the availability of sensitive and specific methods for the rapid and accurate detection of *Pbr*. qPCR has been widely used as a quantitative test for pathogenic bacteria, including *Pectobacterium odoriferum* [35], *Pectobacterium parmentieri* [8], *Pectobacterium carotovorum* ssp. *Carotovorum* [36], and *Pbr* [37]. However, the presence of residual DNA from deceased pathogens can potentially restrict the accurate detection of pathogen numbers via qPCR [37,38,39]. Consequently, this limitation prevents the qPCR technique from effectively serving as an early warning system for pathogenic diseases. Interestingly, PMA can penetrate damaged or dead cell membranes but not intact, living cell membranes [40], which enables irreversible covalent binding to DNA, resulting in its loss during DNA extraction [41]. Based on the *glu* and 23S rRNA nucleic acid sequences in the *Pbr* genome, this study designed the specific detection primers PMA1F/R, which encompass the characteristics of distinguishing *Pectobacterium* from other closely related pathogenic genera [37] and have a single copy in their genome. The qPCR design, which was based on *Pbr* SX309, indicates that the qPCR assay can detect approximately 7 copies·µL^−1^ DNA when the Ct value is less than or equal to 35. The limit of detection observed in this study was at the same level as that observed in previous studies [37]. Furthermore, combining PMA with qPCR as the first attempt to quantify live *Pbr* in bacterial suspensions and contaminated cucumber seeds has important implications for monitoring disease transmission. The limit of detection of the PMA-qPCR assay corresponded to approximately 6 × 10^2^ CFU·mL^−1^. Interestingly, the PMA-qPCR assay system constructed in this study had a higher limit of detection for the number of viable cells in complex sample species compared with a previously reported level of qPCR detection [37], which can be related to the avoidance of the interference of DNA from dead bacteria.

Since its first reported application for the detection of viable cells [26], PMA has been widely employed in the identification of various microorganisms, including bacteria [42,43,44] and fungi [45,46]. Nonetheless, two issues need to be addressed when constructing PMA-qPCR system constructs for the detection of *Pbr*: the lack of amplification of long fragments through the combination of PMA and qPCR [43], and the variability of PMA treatment conditions for different assay strains [26]. Therefore, the target sequence amplified using the specific primers designed in this study was a small fragment (142 bp). Furthermore, this study optimized the conditions for PMA pretreatment of *Pbr* by considering the varying tolerance levels of different strains to PMA. The concentration of PMA was set to 80 μmol·L^−1^, whereas the illumination time was set to 5 min. Consequently, a PMA-qPCR detection system for *Pbr* was successfully developed and had an amplification efficiency of 97% (≥90%). After PMA treatment, the Ct value measured via fluorescence PCR increased with reductions in the percentage of living cells, which was consistent with the results obtained via the plate counting method, whereas the Ct value obtained via ordinary fluorescence quantitative PCR without PMA pretreatment exhibited a smooth change with no significant differences, indicating that this method could not differentiated dead pathogens from living pathogens. Hence, *Pbr* can be detected via the PMA-qPCR assay, and this method effectively prevented the influence of dead cell genomes on the detection results (Table 2). These test results are more in line with the actual probability of disease and can provide an early warning.

The presence of this pathogen in seeds is a significant means through which bacterial soft rot of cucumber is transmitted. This pathogen can effortlessly hide within the seed coat or interior, resulting in an externally “healthy” appearance, which complicates the identification of whether the seed is contaminated and thus makes the prevention and control of this seed-borne disease difficult. Similarly, once the surviving *Pbr* pathogen remains on the surface of potato tubers, it can also induce soft rot symptoms in newly sown potatoes. Although the traditional plate isolation method can isolate and identify pathogenic bacteria from seeds, it is unable to meet the current requirements for detecting and providing early warnings of bacterial soft rot disease, which is primarily due to interference caused by non-pathogenic bacteria and the time and costs involved in identifying pathogenic bacteria using this method [36,43]. Whether the constructed PMA-qPCR detection system can accurately and effectively detect live disease-causing bacteria under practical application scenarios is a criterion reflecting the necessity of system construction. In the current study, whether artificially simulating pathogen immersion or collecting cucumber seeds and potato tubers that may harbor pathogens in different regions, the PMA-qPCR detection system was able to accurately detect pathogens in living cells (Table 2 and Table 3). In addition, the results of the data calculated in this study, based on the PMA-qPCR assay system for live *Pectobacterium brasiliensis*, exhibited higher sensitivity and accuracy compared to the results of plate counting (Table 2 and Table 3), especially when examining complex samples. Plate counts often have a high degree of error due to the bacteria’s extremely similar morphology. Combined with the results obtained from the detection of carrier seeds following artificial infection, this method revealed that the lower limit of detection of viable bacterial counts was approximately 594 CFU·g^−1^ per cucumber seed. This finding indicates the high sensitivity of the PMA-qPCR assay in detecting cucumber seeds and potato tubers carrying the *Pbr* pathogen [43], which emphasizes the significance of early detection of soft rot in cucumbers and potatoes.

Warm broth soaking is a traditional and effective method for controlling seed-borne diseases in agricultural production [47,48,49,50]. While not affecting the normal physiological activity of seeds, the employment of a high-temperature treatment will kill pathogenic organisms carried via seeds and avoid the occurrence of seed-borne diseases. However, residual mycobacterial DNA interferes with the accuracy of traditional qPCR detection; thus, the number of viable pathogens remaining on the seeds can be accurately analyzed via PMA-qPCR and plating counting (Figure 5 and Table S2). The results of this study demonstrated that soaking seeds in hot water at 55~60 °C for 10~30 min effectively killed the live *Pbr* pathogens carried on the seeds (inhibition rate ≥ 90%), which indicates the feasibility of this method [51].

In addition, even if the sown seeds are treated aseptically, the seedlings will still be infected with bacterial soft rot disease due to other factors (such as soil, agricultural tools, or space carrying pathogens). Hence, using simulated cucumber leaves artificially inoculated with the pathogen, we dynamically elucidated the *Pbr* infection status of the plant leaves (Figure 4). After the leaves were infected by the pathogenic bacteria, symptoms began to appear, and the number of pathogenic bacteria increased from 10^4^~10^5^ (CFU·g^−1^) to 10^6^~10^7^ (CFU·g^−1^) (Figure 4). This result is consistent with the plate counting statistics (Table S3). Bacterial soft rot of cucumbers caused via *Pbr* infection can be rapidly diagnosed on plant leaves, even if the symptoms are invisible to the naked eye, and the system provides an early warning of the disease and thus allows aggressive and effective control of the disease. Therefore, PMA-qPCR technology can be employed as an effective tool for early disease diagnosis and warning in cucumber cultivation.

## 5. Conclusions

In this study, the specific primers PMA1-F/R were designed based on the conserved region of the *glu* and 23S rRNA genes in the *Pbr* genome, and a specific PMA-qPCR quantitative detection system for living *Pbr* cells was established for the first time. Moreover, the best PMA pretreatment conditions were determined via optimization: the final concentration was 80 μmol/L. In addition, the number of surviving *P. brasiliensis* was accurately detected in different samples, both artificially simulated as well as naturally collected, based on the constructed PMA-qPCR assay system. In summary, PMA-qPCR is an accurate and efficient method for the quantitative detection of living cells in pathogenic bacterial suspensions and infected seeds. This finding is of great significance for the detection and control of cucumber soft rot caused by *Pbr* infection during the early stages of cucumber production.

## Figures and Tables

**Table 1:** Detection of the specificity of qPCR assays for *Pectobacterium brasiliense* using 48 bacterial strains.

Strain No.	Species	Isolate Code	Host	Geographic Origin	qPCR Test ^a^
1	*Pectobacterium brasiliense*	SX309	Cucumber	Shanxi, China	+
2	*Pectobacterium brasiliense*	B6	Chinese cabbage	Beijing, China	+
3	*Pectobacterium brasiliense*	KC08	Chinese cabbage	Beijing, China	+
4	*Pectobacterium brasiliense*	WBC12	Chinese cabbage	Beijing, China	+
5	*Pectobacterium brasiliense*	WQC3	Celery	Beijing, China	+
6	*Pectobacterium brasiliense*	Y2	Bok choy	Beijing, China	+
7	*Pectobacterium brasiliense*	WYC1	Bok choy	Beijing, China	+
8	*Pectobacterium brasiliense*	HG1501290301	Cucumber	Shanxi, China	+
9	*Pectobacterium brasiliense*	HG1501290302	Cucumber	Shanxi, China	+
10	*Pectobacterium brasiliense*	HG1501290303	Cucumber	Shanxi, China	+
11	*Pectobacterium brasiliense*	HG1501290304	Cucumber	Shanxi, China	+
12	*Pectobacterium brasiliense*	HG1501290305	Cucumber	Shanxi, China	+
13	*Pectobacterium brasiliense*	HG1501290306	Cucumber	Shanxi, China	+
14	*Pectobacterium brasiliense*	HG1501290307	Cucumber	Shanxi, China	+
15	*Pectobacterium brasiliense*	HG1501290308	Cucumber	Shanxi, China	+
16	*Pectobacterium aroidearum*	MTL1911110302	Common calla-lily rhizome	China	−
17	*Pectobacterium aroidearum*	MY2	Konjac	Yunnan, China	−
18	*Pectobacterium aroidearum*	MY7	Konjac	Yunnan, China	−
19	*Pectobacterium aroidearum*	MY10	Konjac	Yunnan, China	−
20	*Pectobacterium aroidearum*	MY10	Konjac	Sichuan, China	−
21	*Pectobacterium carotovorum*	ATCC 15713	Potato	Denmark	−
22	*Pectobacterium carotovorum*	ATCC 39048	Potato	UK	−
23	*Pectobacterium carotovorum*	B2	Chinese cabbage	Beijing, China	−
24	*Pectobacterium carotovorum*	Y13	Bok choy	Beijing, China	−
25	*Pectobacterium carotovorum*	WBC8	Bok choy	Beijing, China	−
26	*Pectobacterium Polaris*	NIBIO1006^T^	Potato	Norway	−
27	*Pectobacterium Polaris*	WBC1	Chinese cabbage	Beijing, China	−
28	*Pectobacterium Polaris*	WBC6	Chinese cabbage	Beijing, China	−
29	*Pectobacterium Polaris*	WBC9	Chinese cabbage	Beijing, China	−
30	*Pectobacterium Polaris*	HYC22041801	Broccoli	Yunnan, China	−
31	*Pectobacterium Polaris*	SC22041801	Lettuce	Yunnan, China	−
23	*Pectobacterium odoriferum*	ATCC 25272	Potato	Unknown	−
33	*Pectobacterium odoriferum*	T4	Chinese cabbage	Beijing, China	−
34	*Pectobacterium odoriferum*	WBC30	Chinese cabbage	Beijing, China	−
35	*Pectobacterium odoriferum*	Q3	Celery	Beijing, China	−
36	*Pectobacterium odoriferum*	WQC17	Celery	Beijing, China	−
37	*Pectobacterium versatile*	BXH21032402	Hydrangea	Yunnan, China	−
38	*Pectobacterium versatile*	Y6	Bok choy	Beijing, China	−
39	*Pectobacterium versatile*	KC-01	Chinese cabbage	Beijing, China	−
40	*Pectobacterium versatile*	KC-03	Chinese cabbage	Beijing, China	−
41	*Pectobacterium versatile*	KC-06	Chinese cabbage	Beijing, China	−
42	*Pseudomonas syringae tomato*	ATCC BAA-871	Tomato	UK	−
43	*Pseudomonas syringae lachrymans*	NM002	Cucumber	Beijing, China	−
44	*Xanthomonas campestris*	ATCC 33913	Cabbage	UK	−
45	*Ralstonia solanacearum*	FQ1112080402	Tomato	Unknown	−
46	*Acidovorax citrulli*	ATCC 29625	Watermelon	USA	−
47	*Clavibacter michiganensis* subsp. *Michiganensis*	ATCC 10202	Tomato	Unknown	−
48	*Erwinia amylovora*	XL2104240301	Pear	Xinjiang, China	−

^a^ +, positive reaction; −, negative reaction.

**Table 2:** Detection of the defined ratio of viable *Pbr* obtained using three different methods.

Ratio of Viable Cells ^a^	qPCR		qPCR-PMA		Colony Counting
	Ct Values	Log_10_ (copies·mL^−1^) ^b^	Ct values	Log_10_ (copies·mL^−1^) ^b^	Log_10_ (CFU·mL^−1^) ^c^
100%	21.71 ± 0.12 a	6.27	20.96 ± 0.17 a	6.49	7.04
50%	22.98 ± 0.11 a	5.89	22.87 ± 0.16 b	5.92	6.79
10%	24.11 ± 0.07 b	5.56	25.43 ± 0.08 c	5.20	6.01
1%	22.34 ± 0.15 a	6.08	27.98 ± 0.21 d	4.42	5.08
0.1%	22.79 ± 0.35 a	5.95	32.21 ± 0.15 e	3.17	4.24
0.01%	25.65 ± 0.13 b	5.11	34.73 ± 0.16 f	2.34	3.31

^a^ The total cell concentration (including viable and dead cells) was approximately 1 × 10^7^ CFU·mL^−1^. ^b^ Logarithmic value of the number of copies·mL^−1^ of the sample to be tested. ^c^ Logarithmic value of the number of viable bacteria per mL of sample to be tested. Note: The data are presented as averages ± SEs, and different lowercase letters indicate significant differences at the 0.05 level.

**Table 3:** Detection of viable *Pbr* in artificially contaminated cucumber seeds.

Inoculating Concentration of *Pbr* (CFU·mL^−1^)	Ct Values	Number of Live Bacteria Per Gram of Cucumber Seeds (CFU·g^−1^) ^a^	Number of Live Bacteria Per Gram of Cucumber Seeds (CFU·g^−1^) ^b^	Bioassay
				DI (%)
1 × 10^8^	23.89 ± 0.32 a	8.49 × 10^5^	9.12 × 10^5^	78.34 ± 1.34
1 × 10^7^	27.13 ± 0.14 b	1.86 × 10^5^	1.62 × 10^5^	53.73 ± 2.81
1 × 10^6^	29.64 ± 0.26 c	1.71 × 10^4^	2.18 × 10^4^	38.58 ± 2.54
1 × 10^5^	32.28 ± 0.17 d	2.85 × 10^3^	4.02 × 10^3^	0.00
1 × 10^4^	34.59 ± 0.28 e	5.94 × 10^2^	4.43 × 10^2^	0.00
1 × 10^3^	>35	-	-	0.00
1 × 10^2^	>35	-	-	0.00
1 × 10^1^	>35	-	-	0.00

^a^ This viable count was calculated from the Ct value after the PMA-qPCR assay. ^b^ The viable bacteria count was calculated via plate counting. Note: The data are presented as the averages ± SEs, and different lowercase letters indicate significant differences at the 0.05 level.

**Table 4:** Detection of *Pbr* on the surface of potato tubers via the PMA-qPCR assay.

Naturally Infested Seed Lots		Ct Values	Concentration (CFU·g^−1^)	Colony Counting (CFU·g^−1^)
Sample No.	Location			
1	Xinjiang, China	31.73 ± 0.45 a	4.14 × 10^3^	5.21 × 10^4^
2	Xinjiang, China	31.41 ± 0.91 a	5.15 × 10^3^	4.42 × 10^4^
3	Xinjiang, China	33.37 ± 1.12 b	1.36 × 10^3^	3.23 × 10^4^
4	Inner Mongolia, China	34.31 ± 0.33 bc	7.19 × 10^2^	1.54 × 10^3^
5	Inner Mongolia, China	33.41 ± 0.59 b	1.32 × 10^3^	4.58 × 10^3^
6	Inner Mongolia, China	33.91 ± 0.13 b	9.43 × 10^2^	2.14 × 10^3^
7	Heilongjiang, China	33.22 ± 0.47 b	1.51 × 10^3^	7.33 × 10^3^
8	Heilongjiang, China	32.85 ± 0.36 ab	1.94 × 10^3^	7.29 × 10^3^
9	Xinjiang, China	>35	-	-
10	Xinjiang, China	>35	-	-
11	Xinjiang, China	>35	-	-
12	Inner Mongolia, China	>35	-	-
13	Inner Mongolia, China	>35	-	-
14	Heilongjiang, China	>35	-	-
15	Heilongjiang, China	>35	-	-

Note: The data are presented as the averages ± SEs, and different lowercase letters indicate significant differences at the 0.05 level.

**Table 5:** Detection of *Pbr* on the surface of cucumber seeds based on the PMA-qPCR assay.

Naturally Infested Seed Lots		Ct Values	Concentration (CFU·g^−1^)	Colony Counting (CFU·g^−1^)
Sample No.	Location			
1	Shandong, China	28.17 ± 0.32 ab	4.64 × 10^4^	1.33 × 10^5^
2	Shandong, China	28.89 ± 0.21 b	2.85 × 10^4^	4.83 × 10^5^
3	Shandong, China	27.39 ± 0.42 a	7.89 × 10^3^	4.31 × 10^4^
4	Liaoning, China	27.32 ± 0.33 a	8.27 × 10^4^	2.93 × 10^5^
5	Liaoning, China	28.55 ± 0.16 ab	3.59 × 10^4^	1.81 × 10^5^
6	Liaoning, China	29.03 ± 0.19 b	2.59 × 10^3^	3.23 × 10^4^
7	Shanxi, China	30.11 ± 0.23 c	1.24 × 10^3^	1.67 × 10^4^
8	Shanxi, China	28.75 ± 0.31 ab	3.13 × 10^4^	5.24 × 10^5^
9	Shanxi, China	28.14 ± 0.28 a	4.74 × 10^4^	4.23 × 10^5^
Other 31 samples		>35	-	-

Note: The data are presented as the averages ± SEs, and different lowercase letters indicate significant differences at the 0.05 level.

## Data Availability

The complete genome sequence of *Pectobacterium brasiliense* SX309 has been deposited in NCBI under the GenBank accession number CP020350.1.

## Supplementary Materials

The following supporting information can be downloaded at: https://www.mdpi.com/article/10.3390/microorganisms11112808/s1, Table S1: Sequence information on the amplification products of primers specific for Pectobacterium brasiliensis; Table S2: The impact of soaking Pbr seeds in warm broth on mortality; Table S3: Dynamic lesions and number of viable cells of Pbr in cucumber and potato leaves from 0 to 48 h after infection with bacterial soft rot.
