# Supplementary material for: Rapid Detection and Quantification of Viable Cells of Pectobacterium brasiliense Using Propidium Monoazide Combined with Real-Time PCR
Source: Microorganisms. 2023 Nov 19;11(11):2808. doi: 10.3390/microorganisms11112808 (PMC10673545; doi:10.3390/microorganisms11112808)
Supplement: Supplementary file 1 [file microorganisms-11-02808-s001.zip › microorganisms-2688445-supplementary.pdf]

Table S1. Sequence information on the amplification products of primers specific for Pectobacterium brasiliensis

Strain No.	Species	Isolate code	Sequences(5′-3′)
1	*Pectobacterium brasiliense*	SX 309	GCGTGCCGGGTTTATGACCTGGTGCGGATTTTTCGTGTCCCCATCGTCTAGAGGCCTAGGACACTGCCCTTTCA CGGCTGTAACAGGGGTTCGAATCCCCTTGGGGACGCCAATCCGATAATGAGTGAAAGACATTATCATT
2	*Pectobacterium brasiliense*	B6	GCGTGCCGGGTTTATGACCTGGTGCGGATTTTTCGTGTCCCCATCGTCTAGAGGCCTAGGACACTGCCCTTTCA CGGCTGTAACAGGGGTTCGAATCCCCTTGGGGACGCCAATCCGATAATGAGTGAAAGACATTATCATT
3	*Pectobacterium brasiliense*	KC08	GCGTGCCGGGTTTATGACCTGGTGCGGATTTTTCGTGTCCCCATCGTCTAGAGGCCTAGGACACTGCCCTTTCA CGGCTGTAACAGGGGTTCGAATCCCCTTGGGGACGCCAATCCGATAATGAGTGAAAGACATTATCATT
4	*Pectobacterium brasiliense*	WBC 12	GCGTGCCGGGTTTATGACCTGGTGCGGATTTTTCGTGTCCCCATCGTCTAGAGGCCTAGGACACTGCCCTTTCA CGGCTGTAACAGGGGTTCGAATCCCCTTGGGGACGCCAATCCGATAATGAGTGAAAGACATTATCATT
5	*Pectobacterium brasiliense*	WQC 3	GCGTGCCGGGTTTATGACCTGGTGCGGATTTTTCGTGTCCCCATCGTCTAGAGGCCTAGGACACTGCCCTTTCA CGGCTGTAACAGGGGTTCGAATCCCCTTGGGGACGCCAATCCGATAATGAGTGAAAGACATTATCATT
6	*Pectobacterium brasiliense*	Y2	GCGTGCCGGGTTTATGACCTGGTGCGGATTTTTCGTGTCCCCATCGTCTAGAGGCCTAGGACACTGCCCTTTCA CGGCTGTAACAGGGGTTCGAATCCCCTTGGGGACGCCAATCCGATAATGAGTGAAAGACATTATCATT
7	*Pectobacterium brasiliense*	WYC 1	GCGTGCCGGGTTTATGACCTGGTGCGGATTTTTCGTGTCCCCATCGTCTAGAGGCCTAGGACACTGCCCTTTCA CGGCTGTAACAGGGGTTCGAATCCCCTTGGGGACGCCAATCCGATAATGAGTGAAAGACATTATCATT
8	*Pectobacterium brasiliense*	HG150 129030 1	GCGTGCCGGGTTTATGACCTGGTGCGGATTTTTCGTGTCCCCATCGTCTAGAGGCCTAGGACACTGCCCTTTCA CGGCTGTAACAGGGGTTCGAATCCCCTTGGGGACGCCAATCCGATAATGAGTGAAAGACATTATCATT
9	*Pectobacterium brasiliense*	HG150 129030 2	GCGTGCCGGGTTTATGACCTGGTGCGGATTTTTCGTGTCCCCATCGTCTAGAGGCCTAGGACACTGCCCTTTCA CGGCTGTAACAGGGGTTCGAATCCCCTTGGGGACGCCAATCCGATAATGAGTGAAAGACATTATCATT

10	*Pectobacterium brasiliense*	HG1501290303	GCGTGCCGGGTTTATGACCTGGTGCGGATTTTTCGTGTCCCCATCGTCTAGAGGCCTAGGACACTGCCCTTTCACGGCTGTAACAGGGGTTCGAATCCCCTTGGGGACGCCAATCCGATAATGAGTGAAAGACATTATCATT
11	*Pectobacterium brasiliense*	HG1501290304	GCGTGCCGGGTTTATGACCTGGTGCGGATTTTTCGTGTCCCCATCGTCTAGAGGCCTAGGACACTGCCCTTTCACGGCTGTAACAGGGGTTCGAATCCCCTTGGGGACGCCAATCCGATAATGAGTGAAAGACATTATCATT
12	*Pectobacterium brasiliense*	HG1501290305	GCGTGCCGGGTTTATGACCTGGTGCGGATTTTTCGTGTCCCCATCGTCTAGAGGCCTAGGACACTGCCCTTTCACGGCTGTAACAGGGGTTCGAATCCCCTTGGGGACGCCAATCCGATAATGAGTGAAAGACATTATCATT
13	*Pectobacterium brasiliense*	HG1501290306	GCGTGCCGGGTTTATGACCTGGTGCGGATTTTTCGTGTCCCCATCGTCTAGAGGCCTAGGACACTGCCCTTTCACGGCTGTAACAGGGGTTCGAATCCCCTTGGGGACGCCAATCCGATAATGAGTGAAAGACATTATCATT
14	*Pectobacterium brasiliense*	HG1501290307	GCGTGCCGGGTTTATGACCTGGTGCGGATTTTTCGTGTCCCCATCGTCTAGAGGCCTAGGACACTGCCCTTTCACGGCTGTAACAGGGGTTCGAATCCCCTTGGGGACGCCAATCCGATAATGAGTGAAAGACATTATCATT
15	*Pectobacterium brasiliense*	HG1501290308	GCGTGCCGGGTTTATGACCTGGTGCGGATTTTTCGTGTCCCCATCGTCTAGAGGCCTAGGACACTGCCCTTTCACGGCTGTAACAGGGGTTCGAATCCCCTTGGGGACGCCAATCCGATAATGAGTGAAAGACATTATCATT

Table S2. The impact of soaking *Pbr* seeds in warm broth on mortality

Treatment time (min)	Mortality rates of *P. brasiliense* in different temperature treatments (%)[a]		
	**50**	**55**	**60**
10	78.2±4.9a	89.6±3.8a	97.3±1.1a
30	89.6±4.2b	95.0±2.0b	100b
60	97.3±5.6c	100c	100b

[a] This result was determined by plate counting. Note: The data are presented as the averages ± SEs.

Table S3. Dynamic lesions and number of viable cells of *Pbr* in cucumber and potato leaves from 0 to 48 hours after infection with bacterial soft rot.

Plant leaves	The number of viable cells of *Pbr* in different time (10^4CFU/g)[a]				
	0h	12h	24h	36h	48h
Potato	3.2±0.1a	21.6±2.2b	73.4±2.4c	168.4±7.1d	421.5±5.4e
Cucumber	0.9±0.1a	6.7±1.3b	21.3±4.1c	98.3±4.8d	165.2±7.2e

[a] This result was determined by plate counting. Note: The data are presented as the averages ± SEs.
